# Periodontal disease and visfatin level: A systematic review and meta-analysis

**DOI:** 10.1371/journal.pone.0293368

**Published:** 2023-11-07

**Authors:** Mojtaba Bayani, MohammadTaha Heidari, Amir Almasi-Hashiani

**Affiliations:** 1 Department of Periodontics, School of Dentistry, Arak University of Medical Sciences, Arak, Iran; 2 Student Research Committee, Arak University of Medical Sciences, Arak, Iran; 3 Department of Epidemiology, School of Health, Arak University of Medical Sciences, Arak, Iran; 4 Traditional and Complementary Medicine Research Center, School of Medicine, Arak University of Medical Sciences, Arak, Iran; Yerevan State Medical University Named after Mkhitar Heratsi, ARMENIA

## Abstract

Visfatin is considered an inflammatory biomarker in periodontal disease (PD). In this meta-analysis, we aimed to evaluate the relationship between Visfatin biomarker level with PD. In this study, Medline, Scopus, Web of Science, and Google Scholar were searched. We included studies that examined visfatin levels in samples from healthy people and periodontal disease until March 2023. The quality of the selected articles was evaluated using the Newcastle-Ottawa assessment scale. Depending on heterogeneity of studies, random-effects or fixed-effect models were used to pool results and report the standardized mean difference (SMD). After screening the retrieved papers, the related data were extracted. A total of 159 studies were identified, and 16 studies were included in the meta-analysis. In 9 studies, the SMD of visfatin level of gingival crevicular fluid (GCF) in patients with chronic periodontitis (CP) and healthy individuals was 4.32 (p<0.001). In 6 studies, the SMD of salivary visfatin level in patients with CP and healthy individuals was 2.95 (p = 0.004). In addition, in five studies, the SMD of serum visfatin level in patients with CP and healthy individuals was 7.87 (p<0.001). Therefore, Visfatin levels in serum, saliva, and GCF of patients with CP were increased in comparison to healthy individuals. Comparison of visfatin levels in saliva of gingivitis patients and healthy individuals showed a significant increase of visfatin in gingivitis patients (SMD:0.57, P = 0.018), but no significant difference was observed in the mean GCF visfatin level of gingivitis patients and healthy individuals (SMD:2.60, P = 0.090). In addition, the results suggested that there is no difference between gingivitis cases compared to CP patients (SMD:3.59, P = 0.217). Visfatin levels in GCF, serum, and saliva have the potential to be used as a diagnostic biomarker of periodontitis.

## Introduction

Periodontal disease is a pathological condition of the periodontium (the supporting structure of the teeth including periodontal ligament, cementum, gingiva, and alveolar bone) [1], which is a set of biological processes including interactions between microorganisms and host immune/ inflammatory responses [2]. Imbalance between these two factors and inappropriate inflammatory responses to pathogenic infections cause loss of connective tissue cohesion and alveolar bone destruction [3]. This local chronic inflammatory process with the destruction of tooth-supporting tissue causes gum bleeding, alveolar bone loss, and dental loss [4]. Local pro-inflammatory cytokines produced in response to bacterial infection mediate periodontal destruction [5]. Currently, the diagnostic standard of periodontal disease is the measurement of clinical indicators such as probing depth, clinical attachment loss (CAL), bleeding on probing, and radiographic examination to check the alveolar bone surface [6]. Inflammatory biomarkers can provide more information in the field of periodontal diseases in addition to standard clinical and radiographic findings [7].

Visceral Fat Adipokine (Visfatin), also known as Pre-B cell colony enhancing factor (PBEF) or Nicotinamide phosphoribosyl transferase (NAMPT), is an adipokine with a molecular weight of 52 kilodaltons and 491 amino acids, which was discovered in 2005. Visfatin is secreted from fat tissue, macrophages, and leukocytes, especially granulocytes and monocytes [8–10] and it increases the production rate of pro-inflammatory and anti-inflammatory cytokines including interleukin 1 beta (IL-1β), interleukin-1 receptor antagonist (IL-1Ra), interleukin 6 (IL-6), interleukin 10 (IL-10), and tumor increases necrosis factor alpha (TNF-α) in human monocytes [11]. In human bone marrow, it is a cytokine-like molecule that stimulates the early stages of B cell formation [12]. It is also secreted by neutrophils in response to pathogens and stimulates monocytes to produce inflammatory mediators [11].

In general, visfatin expression increases in inflammatory conditions [13]. In periodontal diseases as an inflammatory condition, an increase in visfatin levels in saliva, serum, gingival crevicular fluid (GCF) and gum tissue has been reported in various studies [14–19]. According to the results of these studies, visfatin can be used as an inflammation biomarker in periodontal diseases [16,20,21].

Since the need to investigate the inflammatory process of periodontal diseases concerning visfatin biomarker has been noted and several studies have determined the relationship between visfatin and periodontal disease and there are several studies in this field that have examined the level of visfatin in patients with periodontitis and healthy people, but their results have been contradictory and there is no comprehensive study that summarizes the findings of previous studies, this study aims to conduct a systematic review and meta-analysis to comprehensively clarify the importance of visfatin biomarker in periodontal diseases and its diagnostic role. Therefore, the main purpose of this systematic review and meta-analysis is to investigate the expression of visfatin biomarker in periodontal diseases.

## Materials and methods

### Study design

This study is a systematic review and meta-analysis, which was conducted based on Preferred Reporting Items for Systematic Reviews and Meta-Analyses (PRISMA) [22].

### Search strategy

Medline (Via PubMed), Web of Science, and Scopus databases were reviewed according to the search strategy and keywords to identify studies until March 12, 2023. Google Scholar was used to find gray literatures. A manual search was also performed in the references of included studies by MTH. The searched keywords were as follows: Periodontal Diseases, Periodontitis, Gingivitis, Periodontal Disease, Periodontal Index, Nicotinamide Phosphoribosyl transferase, and visfatin. The searches were conducted by two authors (AAH and MTH). The details of the full search strategy in databases are shown in **S1A–S1C Table**.

### Inclusion and exclusion criteria

The search strategy was prepared based on Population/Problem, Exposure/Intervention, Comparison/Control, and Outcome (PICO). The interested population in the search strategy was cases with periodontitis (population) and they were compared with healthy people (comparison) as control group in terms of visfatin level (outcome) of GCF, serum, and saliva. The studies that met the inclusion criteria were those with observational, cross-sectional, case-control, and cohort study designs. Furthermore, clinical trials that had relevant baseline data were also considered. Only studies in English that were published in journals or presented at congresses were included, and studies examined visfatin levels in GCF, serum, and saliva samples of both healthy people (control group) and people with periodontal disease. The evaluation of cases with periodontal disease was based on the gold standard of periodontal clinical evaluation including periodontal pocket probe depth (PPD) and the measurement of CAL [6]. There was no restriction on the gender and age of the study subjects. Any studies that were conducted in vitro or on animals were excluded, as well as those without relevant data and those for which the full text in English was unavailable.

### Study selection and data collection process

After completing the search, the retrieved studies were selected in two stages using Endnote 10 software. Two authors (MB and MTH) screened the studies, and any disagreements were resolved through discussion by the authors and ultimately MB declared the final decision. For this purpose, duplicated articles were first removed, then the title and abstract of remaining studies were checked, and irrelevant studies were removed, In the second stage, the full text of the remaining articles was screened for inclusion and exclusion criteria and eligible studies were included. The data were extracted by two authors (MTH and MB). An email was sent to the corresponding authors of studies whose full text was not available or in cases where related data were not reported in the article.

### Data items

From the included studies, the first author, the year of publication, the country, the number of study subjects, including those with periodontal disease and healthy subjects as the control group, the age of the subjects, the method of measuring visfatin levels, the type of study, the quality score, and the main conclusions, including the mean of Visfatin concentration levels (ng/ml) and standard deviation were extracted in the group with periodontal disease and healthy group.

### Risk of bias in individual studies

The quality of the selected articles was evaluated using the Newcastle-Ottawa assessment scale [23], which was adapted for Case-control and Cohort studies in the form of two separate checklists, and the score range of this checklist is from zero to nine. Finally, the articles were classified in terms of quality and placed in three levels: low quality with a score of less than 3, medium quality with a score between 3 and 6, and high quality with a score of more than 6. The risk of bias assessment was done by MTH under the supervision of AAH.

### Statistical analysis

To assess the heterogeneity between included studies, Chi-square test and I-square statistic were used and I-square above 50% was measured as substantial heterogeneity. Based on the heterogeneity analysis, in cases where there was significant heterogeneity, the random-effects model was used and in cases where the heterogeneity was low, the fixed-effect model was used to pool the results. Funnel plot and Egger’s linear regression were used to investigate the existence of publication bias. Considering that in this meta-analysis comparison of means was used, the results are expressed as standardized mean difference (SMD). The extracted data were first entered into Excel software and after adjustment and finalization, all statistical analyses were done by Stata software version 16 (Stata Corp, College Station, TX, USA).

## Results

### Study selection and characteristics

**Fig 1:**
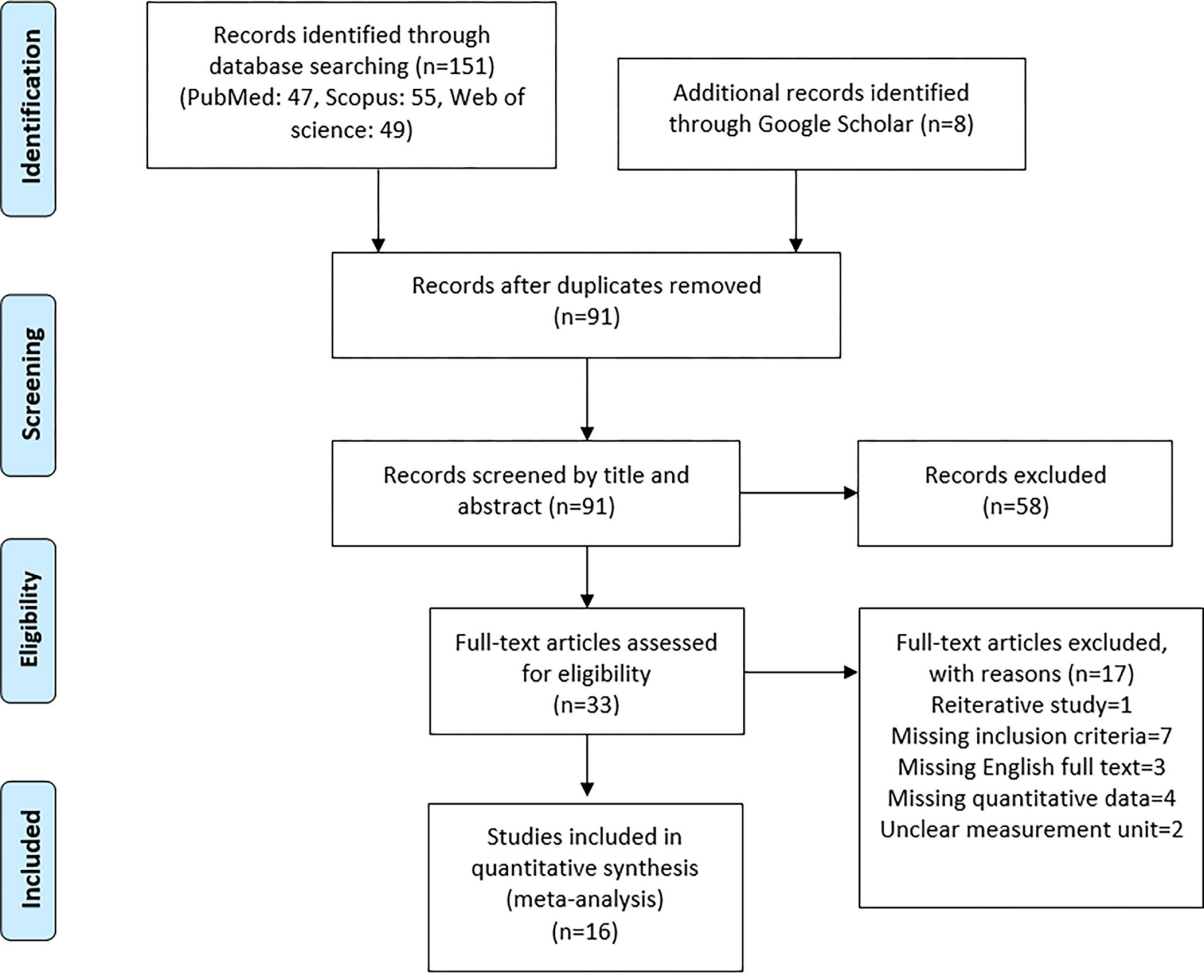
Flow diagram of the literature search for studies included in meta-analysis.

**Fig 1** shows the process of identifying studies, removing duplicates, and screening based on title, abstract, and full text of studies. After searching the databases, a total of 159 studies (PubMed: 47, Scopus: 55, Web of Science: 49, and Google Scholar: 8) were identified. After removing duplicates, 91 studies remained for screening. After screening the title and abstract of these studies by two authors independently, 58 studies that did not meet the inclusion criteria were excluded and the full text of the remaining 33 studies was examined. Finally, sixteen studies [16,17,19,24–36] were included in the meta-analysis. Seventeen studies that were impossible to include in the meta-analysis are presented in **S2 Table** with details and reasons [14,15,18,37–50] and can be considered as qualitative data. In most studies, periodontal diseases were classified based on the 1999 classification for periodontal diseases and conditions [51] and 4 studies [34,47–49] based on the 2018 classification [52]. One of the studies analyzed biopsy samples, one studied saliva, one studied saliva and serum without providing quantitative data, and one studied GCF. Salivary visfatin concentration of healthy and gingivitis groups from one of them could used in our meta-analysis. Four [14,18,37,48] studies that examined visfatin expression in tissue biopsies should be excluded but we investigated them so their result could not pooled into a meta-analysis.

The sample size of included studies in the meta-analysis ranged from the lowest number of 19 cases to the highest number of 60 cases, including both sexes. The age of participants was youth and middle-aged. The oldest study is related to 2011 and the most recent is related to 2022. The characteristics of the included studies are shown in **Table 1**.

**Table 1:** A summary of the included study in meta-analysis characteristics.

ID	Author	Year	Country	Study	Age	Case group	Visfatin analysis method	Control visfatin level, means (SD)	Case visfatin level, means (SD)	Risk Score
1	Pradeep AR [16]	2011	India	cross-sectional study	30.08	Gingivitis, Chronic periodontitis	Serum (ELISA), (ng/mL) GCF (ELISA) (ng/mL)	7.96 (1.1), 10.85 (2.62)	G.S: 19.75 (2.08), CP.S: 57.62 (7.34), G.GCF: 31.23 (5.85), CP.GCF: 72.78 (6.75)	High
2	Pradeep AR [17]	2012	India	cross-sectional study	32.96	Chronic periodontitis	Serum (ELISA), (ng/mL) GCF (ELISA) (ng/mL)	8.02 (0.98), 11.16 (2.32)	52.48 (5.87), 71.41 (6.26)	High
3	Raghavendra NM [24]	2012	India	RCT	35.06	Chronic periodontitis	Serum (ELISA), (ng/mL) GCF (ELISA) (ng/mL)	8.398 (1.047), 11.49 (2.297)	58.97 (6.845), 70.04 (7.257)	High
4	Banna SE [25]	2013	Egypt	cross-sectional study	23–50	Gingivitis, Chronic periodontitis	GCF(ELISA) (ng/mL)	6.7 (7.4)	G: 44.1 (42.2) CP: 73.99 (37.23)	Moderate
5	Tabari ZA [19]	2014	Iran	cross-sectional	36.15	Chronic periodontitis	Saliva (ELISA) (ng/mL)	23.38 (7.58)	33.43 (15.72)	High
6	Kadkhodazadeh M [26]	2016	Iran	cross-sectional study	40.14	Chronic periodontitis, Peri-implantitis	Saliva (ELISA) (ng/mL)	23.97 (10.35)	CP: 11.95 (14.28), PI: 12.83 (11.11)	Moderate
7	Ozcan E [27]	2016	Turkey	RCT	42.71	Chronic periodontitis	Saliva (ELISA) (ng/mL)	2.56 (0.68)	3.48 (1)	High
8	Cetiner D [28]	2019	Turkey	RCT	41.58	Chronic periodontitis	GCF(ELISA) (picogram/mL)	7.15 (3.12)	10.65 (5.72)	Moderate
9	Mopidevi A [29]	2019	India	RCT	44	Chronic periodontitis	Saliva (ELISA) (ng/mL)	27.33 (7.19)	45.96 (7.0879)	High
10	Rezaei M [30]	2019	Iran	cross-sectional study	45.01	Chronic periodontitis	GCF (ELISA) (picogram/mL)	12.59 (2.95)	23.15 (3.17)	High
11	Surya D [31]	2019	India	RCT	42.44	Chronic periodontitis	Serum (ELISA), (ng/mL) Saliva (ELISA) (ng/mL)	16.423 (2.11), 19.943 (2.13)	38.1 (2.89), 57.86 (3.35)	High
12	Paul R [32]	2020	India	cross-sectional study	30–55	Chronic periodontitis	GCF (ELISA) (ng/mL)	0.43 (0.11)	0.95 (0.23)	High
13	Saljoughi F [33]	2020	Iran	case-control study	45.37	Chronic periodontitis	GCF (ELISA) (picogram/mL)	13.15 (2.95)	21.54 (3.17)	High
14	Coutinho A [34]	2021	India	cross-sectional study	32.21	Gingivitis, Periodontitis	Saliva (ELISA) (ng/mL)	25.6 (2.19)	G: 26.66 (2.24), P: 38.22 (3.38)	High
15	Saseendran G [35]	2021	India	RCT	25–50	Gingivitis, Chronic periodontitis	Saliva (ELISA) (ng/mL)	19.2 (1.9)	G: 20.9 (2.9), CP: 24.8 (2.1)	High
16	Xu T.-H [36]	2022	China	cross-sectional study	43	Chronic periodontitis	Serum (ELISA) (ng/ul) GCF (ELISA) (pg/ul)	16.15 (5.09) 4.71 (2.60)	33.87 (10.86) 4.52 (1.80)	High

Note: Coutinho A’s [34] study classified periodontal disease based on the 2018 classification and others are based on the 1999 classification. SD = standard deviation; GCF = Gingival Crevicular Fluid; ELISA = enzyme-linked immunosorbent assay; G.S = Gingivitis. Serum; CP.S = Chronic periodontitis. Serum; G.GCF = Gingivitis. Gingival Crevicular Fluid; CP.GCF = Chronic periodontitis. Gingivitis. Gingival Crevicular Fluid; RCT = Randomized controlled trial; G = gingivitis; CP: Chronic periodontitis; PI: Peri-implantitis; P: Periodontitis.

### Risk of bias within studies

The quality of the retrieved studies was evaluated by Newcastle-Ottawa assessment scale and the results were reported as risk scores in **Tables 1 and S2**. A full detailed Newcastle-Ottawa assessment scale is presented in **S3 Table**. Included studies in meta-analysis were categorized in terms of quality, and there were no studies with low quality, 18.75% of studies (n = 3) were medium quality and 81.25% (n = 13) were high quality.

### Comparison of visfatin level in GCF of gingivitis patients and healthy individuals

Only in two studies [16,25], visfatin levels in GCF were compared between gingivitis patients and healthy individuals, and 20 healthy cases and 30 gingivitis patients were included in the analysis, and SMD was estimated as 2.60 ng/ml (95% confidence interval (CI): -0.40–5.62, p = 0.090) (**Table 2**). The results of the present study showed that the visfatin level in the GCF of gingivitis patients and healthy individuals are the same. The analysis of heterogeneity suggested evidence of considerable heterogeneity between studies (I^2^ = 92.1% and P<0.001), therefore, a random-effects model was used to pool the primary results.

**Table 2:** Summary of meta-analysis results and subgroups analysis.

Groups	No of studies	Test of comparison				Heterogeneity		
		SMD (95% CI)	P value	Model	Z	Chi square	P value	I square (%)
GCF visfatin (Gingivitis individuals vs control)	2	2.60 (-0.40–5.62)	0.090	Random	1.69	12.72	<0.001	92.1
Saliva visfatin (Gingivitis individuals vs control)	2	0.57 (0.10–1.04)	0.018	Fixed	2.38	0.20	0.658	0.00
GCF visfatin (Chronic periodontitis individuals vs control)	9	4.32 (2.71–5.93)	<0.001	Random	5.25	168.38	<0.001	95.2
Serum visfatin (Chronic periodontitis individuals vs control)	5	7.87 (3.51–12.23)	<0.001	Random	3.54	113.7	<0.001	96.5
Saliva visfatin (Chronic periodontitis individuals vs control)	6	2.95 (0.96–4.95)	0.004	Random	2.90	137.08	<0.001	96.4
GCF visfatin (Gingivitis individuals vs Chronic periodontitis)	2	3.59 (-2.11–9.30)	0.217	Random	1.236	32.36	<0.001	96.9

Note: GCF = Gingival Crevicular Fluid; SMD = standardized mean difference; CI: Confidence Interval.

### Comparison of visfatin level in saliva of gingivitis patients and healthy individuals

In two studies [34,35], saliva visfatin levels were compared between gingivitis patients and healthy individuals consisting of 36 healthy cases and 36 gingivitis patients were included. The results of the meta-analysis suggested SMD = 0.57 ng/ml (95%CI. 0.1–1.04, P = 0.018) (**Table 2**), which shows that the visfatin level in the saliva of gingivitis patients and healthy individuals are significantly different. The results of chi-square test showed no evidence of heterogeneity and the fixed-effects model was used to merge the data (I^2^ = 0.0%, P = 0.658). The results of the present study showed that the level of visfatin in the saliva of gingivitis patients is higher in comparison to healthy people.

### Comparison of visfatin level in GCF of chronic periodontitis patients and healthy individuals

**Fig 2:**
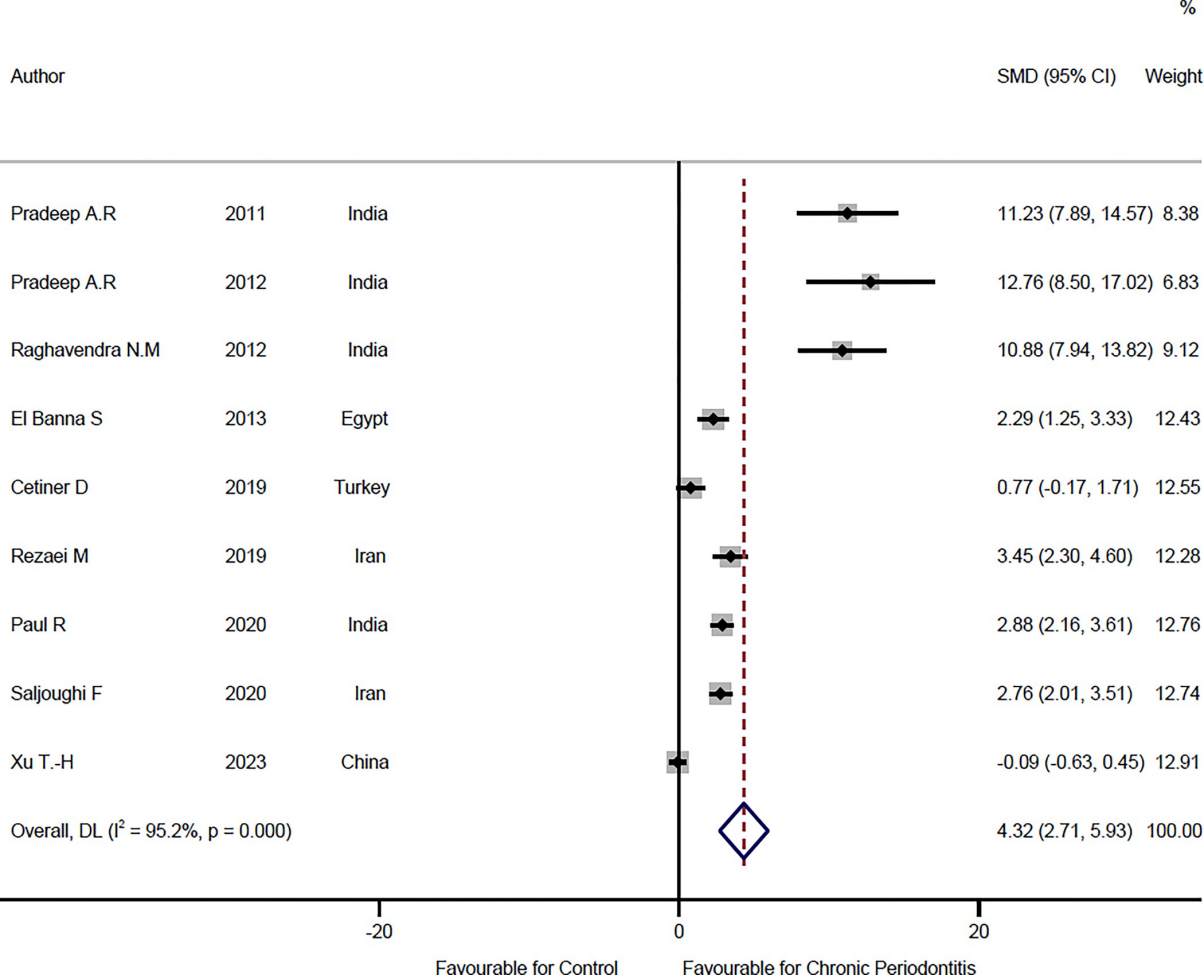
Forest plot comparing the visfatin level in gingival crevicular fluid of chronic periodontitis patients and healthy individuals.

**Fig 3:**
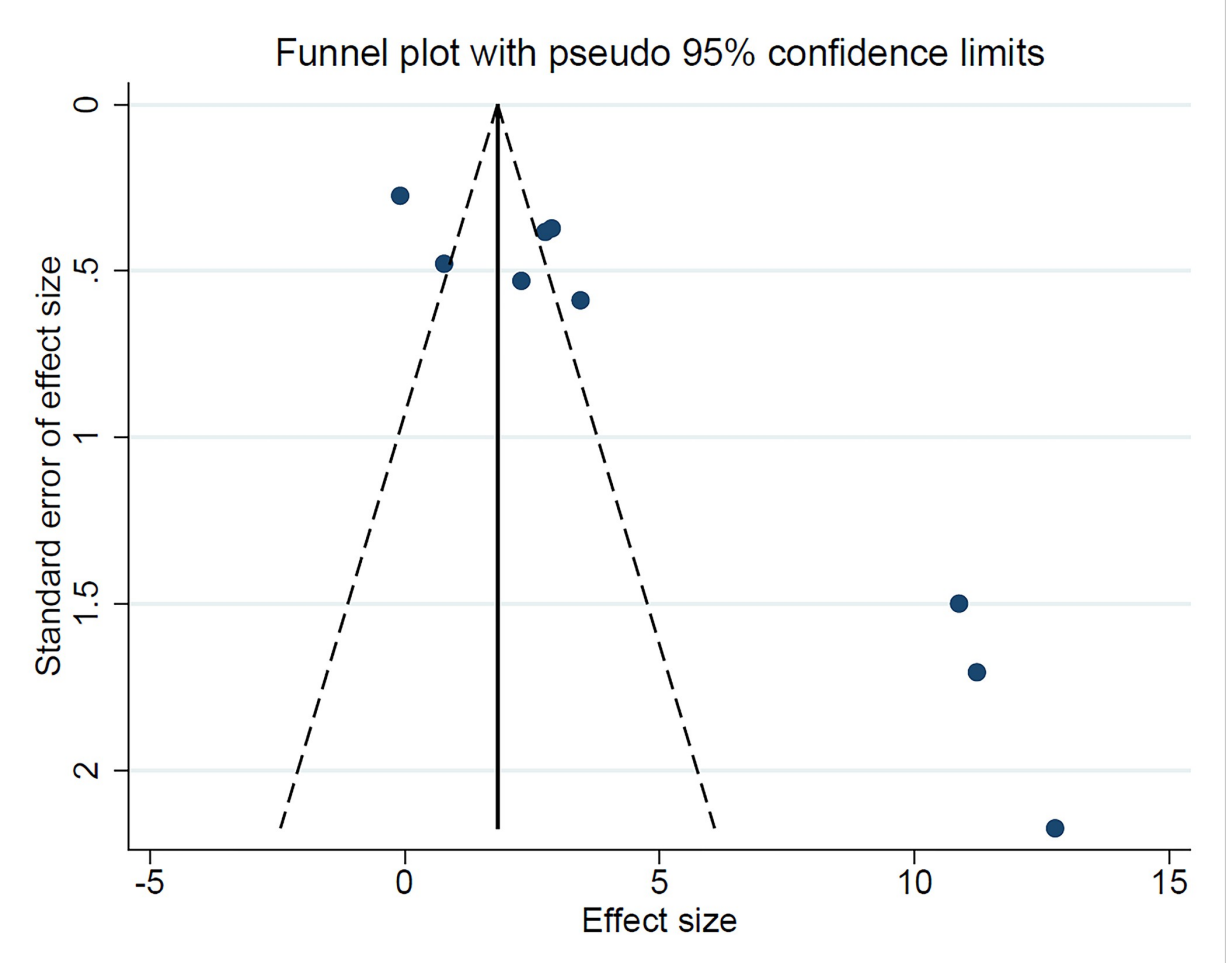
Funnel plot to check the publication bias.

In nine studies [16,17,24,25,28,30,32,33,36], visfatin level of GCF was compared between chronic periodontitis patients and healthy individuals, which includes 152 healthy cases and 172 chronic periodontitis patients. The results of the meta-analysis revealed that SMD = 4.32 ng/ml (95% CI: 2.71–5.93, P<0.001) (**Fig 2, Table 2**) so Visfatin levels in GCF were significantly higher in periodontitis in comparison to the control group. Since there is significant heterogeneity between studies (I^2^ = 95.2% and P<0.001), the random-effects model was used. The funnel plot of this analysis (**Fig 3**) shows the asymmetry and the possibility of publication bias. Also, the result of the Egger test (t = 4.34, P = 0.003) shows evidence of publication bias.

### Comparison of serum visfatin levels in chronic periodontitis patients and healthy individuals

**Fig 4:**
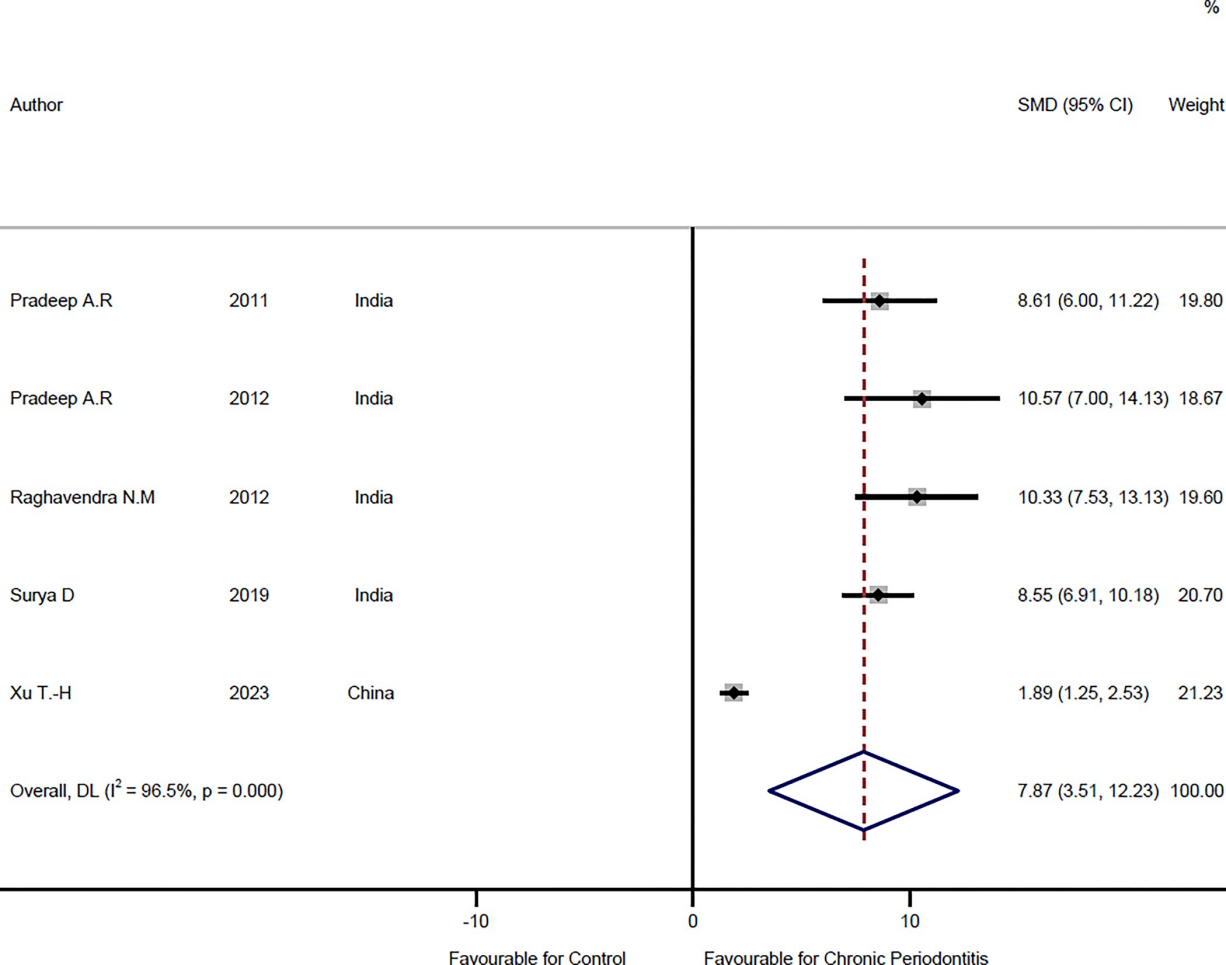
Forest plot comparing the serum visfatin levels in chronic periodontitis patients and healthy individuals.

In five studies [16,17,24,31,36] serum visfatin levels were compared between chronic periodontitis patients and healthy individuals, which includes 85 healthy cases and 115 chronic periodontitis patients. The meta-analysis results of this comparison suggest SMD = 7.87 ng/ml (95%CI: 3.51–12.23, P<0.001) (**Fig 4, Table 2**). Visfatin levels in serum were significantly higher in chronic periodontitis in comparison to the control group. Based on the chi-square test, there was evidence of heterogeneity between studies (I^2^ = 96.5%, P = 0.001), and the random-effects model was used to pool the results. Moreover, the funnel plot and Egger’s tests (t = 4.78, P = 0.017) show evidence of publication bias.

### Comparison of salivary visfatin levels of chronic periodontitis patients and healthy individuals

**Fig 5:**
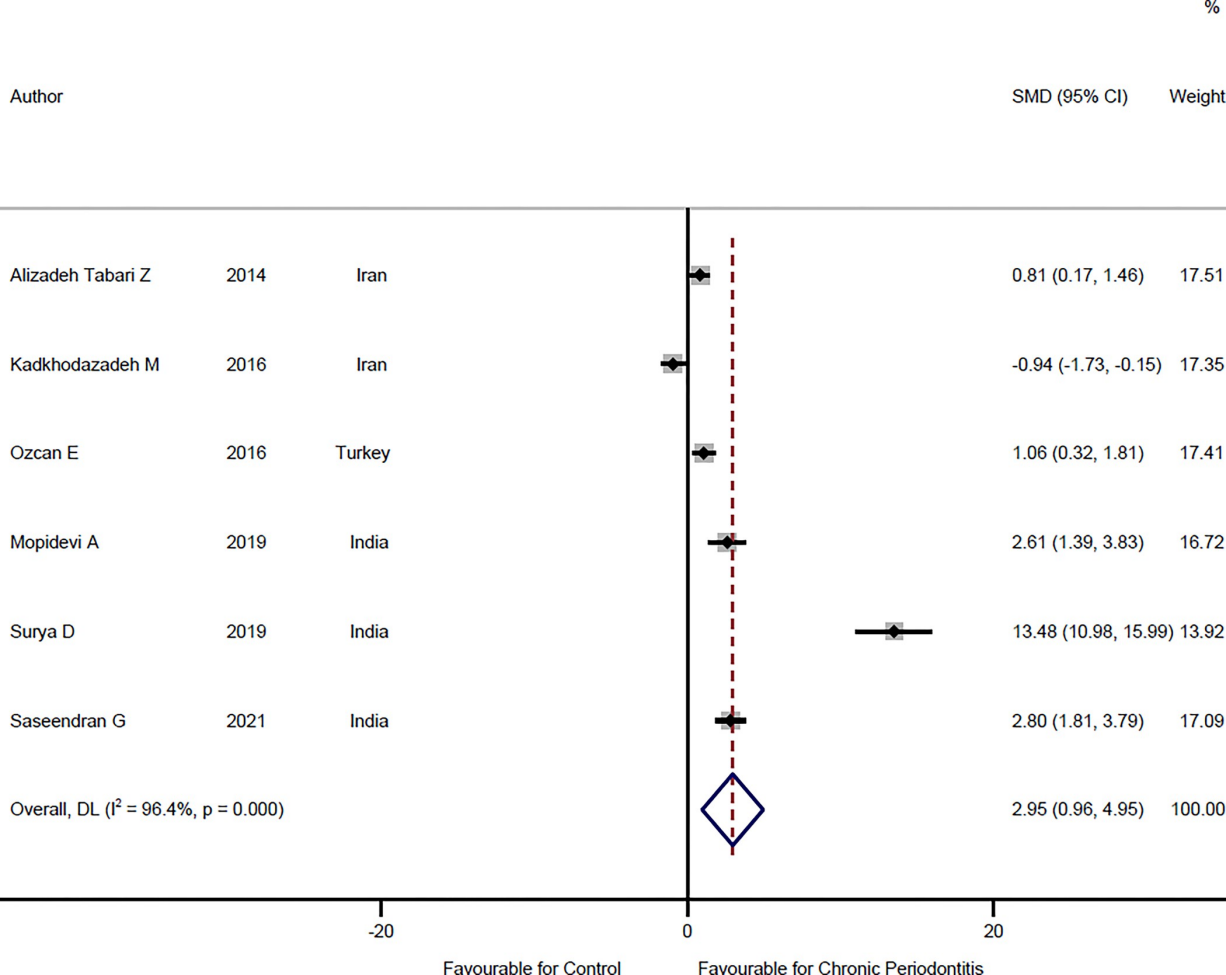
Forest plot comparing the salivary visfatin levels of chronic periodontitis patients and healthy individuals.

In six studies [19,26,27,29,31,35] saliva visfatin levels were compared between chronic periodontitis patients and healthy individuals. This analysis includes 103 healthy cases and 109 chronic periodontitis patients. The results of meta-analysis suggested an SMD = 2.95 ng/ml (95%CI: 0.96–4.95, P = 0.004) (**Fig 5, Table 2**). The results of the present study showed that the level of visfatin in the saliva of chronic periodontitis patients is higher in comparison to healthy individuals. The heterogeneity assessment showed that there is significant heterogeneity between the studies, therefore, the random-effect model was used to pool the results (I^2^ = 96.4%, P<0.001). To assess the publication bias, the funnel plot and Egger test (t = 3.44, P = 0.026) show the asymmetry and the possibility of publication bias.

### Comparison of visfatin level in GCF of gingivitis patients and chronic periodontitis patients

Only in two studies [16,25], visfatin levels in GCF were compared between gingivitis patients and chronic periodontitis patients, and 30 gingivitis patients and 30 chronic periodontitis patients were included in the analysis, SMD was estimated as 3.59 ng/ml (95%CI: -2.11–9.30, p = 0.217) (**Table 2**). The results of the present study showed no significant visfatin level differences in the GCF of gingivitis patients and chronic periodontitis patients. The analysis of heterogeneity suggested evidence of considerable heterogeneity between studies (I^2^ = 96.9% and P<0.001), therefore, a random-effects model was used to pool the primary results.

## Discussion

In this meta-analysis, sixteen studies were included in the analysis. The most important finding of this study showed that the mean level of GCF, saliva, and serum visfatin in chronic periodontitis patients significantly increases compared with healthy individuals. Comparison of visfatin level in saliva of gingivitis patients and healthy individuals showed a significant increase of visfatin in gingivitis patients, but no significant difference was observed in the mean of GCF visfatin of gingivitis patients and healthy individuals. The comparison of visfatin between the two groups of gingivitis patients and healthy individuals was investigated in only two studies, which requires more preliminary studies for precise conclusions. There were no significant differences in visfatin levels between patients with gingivitis and chronic periodontitis, according to GCF analysis.

In the pathogenesis of periodontal diseases, various pro-inflammatory cytokines play a decisive role [53]. For example, TNF-alpha, IL-6, and prostaglandin E2 (PGE2) increase the activity of matrix metalloproteinase (MMP) in the periodontium and ultimately cause soft tissue and alveolar bone degeneration. Finally, these cytokines enter the GCF and reach to the saliva [35]. Nowadays, these mediators are used as biomarkers to diagnose and evaluate the activity of periodontal diseases. Visfatin as a pro-inflammatory adipocytokine is secreted from visceral adipose tissue and neutrophils and macrophages, which increases the secretion of TNF-alpha and IL-6 [10,54]. Visfatin increases the life of macrophages, prevents the apoptosis of neutrophils [55], and induces the expression and activity of MMP [56]. The role of visfatin as an inflammatory biomarker in various diseases such as diabetes [13], inflammatory bowel disease [11], rheumatoid arthritis [57], and cardiovascular diseases [58] has been clarified. Several studies have explored the role of visfatin in periodontal diseases. These studies have examined the visfatin levels in different samples using various analysis methods such as enzyme-linked immunosorbent assay in serum, GCF, and saliva, polymerase chain reaction (PCR), and immunohistochemistry assay of biopsy samples in all types of periodontal diseases. Most studies compared chronic periodontitis individuals with healthy controls. Studies also examined individuals with gingivitis, periodontitis according to the 2017 workshop classification, peri-implantitis, and aggressive periodontitis.

Over the past 30 years, there have been several revisions to the classification of periodontitis. Based on the current understanding in the field of pathophysiology, the 2017 workshop identified three forms of periodontitis: necrotizing periodontal disease, periodontitis as a manifestation of systemic diseases, and forms of the disease previously known as chronic or aggressive (localized and generalized) periodontitis, it is now classified as a single group under the title of periodontitis [52]. So, the phrase "periodontitis" can refer to chronic periodontitis and other related conditions.

There are some advantages of Visfatin as a diagnostic biomarker of periodontitis in comparison to other cytokines. In comparison with other cytokines, it is also possible to measure visfatin level through saliva and GCF, which is an important advantage compared to cytokines that can only be measured through serum samples. And it is probably easier to measure, less invasive and less expensive.

### GCF

The results of our study showed that the visfatin level of GCF in patients with periodontitis is significantly higher in comparison to healthy individuals. Considering the inflammatory nature of periodontitis and the role of visfatin in the inflammatory process, and pointing out that GCF is in full contact with the periodontal tissue and can reflect the conditions of that tissue [20], this increase is justified. On the other hand, the difference in the mean visfatin level of GCF of people with gingivitis disease was not significantly higher compared to healthy people and an important point is that this result contradicts the results of the included studies and other studies that were identified during the systematic review.

Pradeep AR et al. [16,17], states that the increase in visfatin is directly related to the progress and severity of periodontal disease based on clinical parameters. In their study of visfatin relationship with periodontal health and disease [16] in which there were three groups: healthy, gingivitis, and chronic periodontitis, they concluded that the level of visfatin in gingivitis patients was higher than in healthy cases and chronic periodontitis was higher than both gingivitis patients and healthy cases. They introduce visfatin as a biomarker of periodontal disease. Banna SE et al. also confirmed the relationship between the severity and progression of periodontal disease with visfatin levels. In their study, visfatin level was higher in chronic periodontitis and gingivitis than in healthy subjects, but the difference between the chronic periodontitis group and gingivitis group was not statistically significant [25] as we did. Turer CC et al. [43] also supposed that the level of visfatin in patients with gingivitis was higher than in healthy patients, and chronic periodontitis was higher than in both cases and in fact, the concentration of visfatin was proportional to the severity of the inflammation of the disease. They confirm that visfatin can be a biomarker of periodontal disease. Bahammam MA et al [45] demonstrated periodontal destruction and diabetes have a synergistic effect on the elevation of GCF inflammatory cytokine levels as visfatin. However, in the study by Xu T.-H et al [36], the concentration of visfatin in GCF among healthy, chronic periodontitis, and chronic periodontitis with type 2 diabetes groups had no statistical significance. However, the total amount of GCF visfatin in healthy group was significantly lower compared to other two groups. Paul R et al [32] evaluated the association between Porphyromonas gingivalis and visfatin levels in chronic periodontitis patients. They noted a significant positive correlation between GCF visfatin levels with clinical periodontal parameters and visfatin can demonstrate periodontal destruction and the load of inflammation. Also, in the study by Ozcan E et al [42], there was a positive correlation between the GCF visfatin levels and Porphyromonas gingivalis and clinical periodontal parameters. They pointed out that the reason for visfatin increase could be related to the periodontal microbiome. The point is GCF flow increases in inflammatory conditions, which can lead to an increase in the level of visfatin. However, in various studies, there was a significant difference in visfatin levels between individuals with periodontitis and healthy people. This suggests that the dominant cells in periodontal disease activity are responsible for the increase in visfatin release. Shalaby HK et al [47] study was the only one investigating GCF visfatin in periodontitis based on the 2017 classification. They figured that higher visfatin levels in periodontitis patients than in healthy individuals and this visfatin increase is related to the severity of the disease. In addition, the role of visfatin in different stages of periodontal disease process was suggested as the reason for the variation of visfatin concentration in the GCF at the time of sampling.

Variations in GCF flow and different methods of collecting GCF or time of sampling can contribute to data heterogeneity, with the most common method using micropapillary peptide and paper strips.

A small number of studies have compared GCF visfatin levels of gingivitis with healthy subjects and with periodontitis. So, it is suggested that more studies should be conducted with a full comparison of different disease groups with healthy people and more participants.

### Serum

Cytokine activity of the body is usually evaluated by measuring their serum level [59] and since serum components show the level of the host’s inflammatory response to periodontal disease pathogens [60], it is possible to use the serum level of visfatin cytokine to investigate the periodontal disease process. In this meta-analysis, it was clarified that the serum visfatin level of people with chronic periodontitis is significantly higher than healthy people, this result is in accordance with the results of the previous studies.

Pradeep AR et al. found that the serum visfatin level increases according to periodontal disease severity. In their study, the mean serum visfatin level in gingivitis patients was higher in comparison to healthy individuals. They considered serum visfatin, like GCF visfatin, as an inflammatory biomarker of periodontal disease [16,17]. In the study by Raghavendra NM et al, the increase in visfatin levels in chronic periodontitis compared to healthy individuals was interpreted as a sign of the role of visfatin in the pathogenesis of periodontal diseases [24]. Turer CC et al also reported an increased level of serum visfatin in patients with chronic periodontitis compared to gingivitis and both groups compared to healthy individuals and suggested visfatin as an inflammatory biomarker of periodontal disease [43]. Xu T.-H et al [36] reported lower serum visfatin level in healthy group than chronic periodontitis group and chronic periodontitis with type 2 diabetes group. Furthermore, serum visfatin in chronic periodontitis group was significantly lower than in chronic periodontitis with type 2 diabetes group. They demonstrated an inflammatory link between periodontitis and type 2 diabetes mellitus. They found a positive correlation between fasting blood glucose and serum visfatin level. Kemer Doğan et al [49] supposed positive relationships between salivary and serum visfatin levels with periodontal parameters and with obesity. They classified periodontitis cases and controls based on the 2017 periodontal classification. Inflammatory conditions of individuals, blood glucose, and obesity can lead to data heterogeneity.

Serum visfatin can be considered as an inflammatory biomarker of periodontitis and gingivitis these show visfatin activity in the pathogenesis of periodontal disease. On the other hand, considering that the increased concentration of serum visfatin is known as a risk factor for cardiovascular diseases [44,58] and considering the inflammatory link between blood glucose, obesity, and visfatin, it is crucial to remember the significance of visfatin activity in the pathogenesis of the disease.

### Saliva

Saliva is a biological fluid that can be collected non-invasively and without pain and special equipment. On the other hand, saliva contains local and systemic indicators of periodontal diseases, and these inflammatory indicators can be easily evaluated in saliva [61]. In this study, the level of salivary visfatin of people with periodontal disease and healthy people was compared. The result of this meta-analysis shows the increased level of salivary visfatin in people with chronic periodontitis compared to healthy people.

In the study of Kadkhodazadeh M et al [26], the salivary level of visfatin was compared in three groups: healthy, chronic periodontitis, and peri-implantitis and no significant difference was reported between the salivary visfatin levels of these groups. They declared difficulty of clinical diagnosis of initial gingivitis from healthy individuals and unreliable visfatin measurement in saliva may cause contrary results. While Ozcan E et al [27] and Saseendran G. et al [35] considered the increase of salivary visfatin proportional to the severity of the disease. They point out that the role of visfatin in the pathogenesis of periodontal disease has been determined and, in their results, they introduced salivary visfatin as a reliable biomarker for evaluating the results of periodontal disease treatment. Ozcan E et al [27] and Omer B et al [41] found a positive correlation between salivary visfatin level and both CAL and probing depth. In contrast, Tabari ZA et al [19] found that only CAL was related to salivary visfatin levels. Also, Ozcan E et al [15] supposed that the level of salivary visfatin in patients with gingivitis and chronic periodontitis was higher than in healthy individuals. However, they did not find any significant difference between the groups of chronic periodontitis and gingivitis. The researchers considered salivary visfatin as a periodontal inflammatory biomarker, but they also noted that it was incomplete in distinguishing between gingivitis and periodontitis. The results of Coutinho A. et al [34] and Saseendran G. et al [35] studies which were meta-analyzed and Ozcan E. et al [27] showed that the level of visfatin in the saliva of gingivitis patients is higher in comparison to healthy people which is consistent with our findings. Two studies investigated periodontal disease based on the 2017 periodontal classification. Coutinho A et al [34] showed higher salivary visfatin concentrations in patients with gingivitis and periodontitis compared with periodontally healthy individuals and a negative correlation between Body mass index (BMI) and salivary visfatin levels. Kemer Doğan et al [49] figured higher salivary visfatin levels in periodontitis groups are correlated with periodontal parameters and obesity.

In general, salivary visfatin is proposed as an inflammatory biomarker of periodontitis and gingivitis, which plays a role in the pathogenesis of periodontal diseases, and can also have a diagnostic role.

In this systematic review, we encountered 4 studies that examined visfatin and mRNA expression levels in periodontal tissues through biopsy. However, due to the limited number of studies, the variety of periodontal disease classifications, the different methods of measurement, and the lack of quantitative data, meta-analysis was not possible. A summary of their results is shown in **S2 Table**. They all concluded that higher visfatin levels are associated with periodontitis.

One of the limitations of this study was data heterogeneity and possible publication bios. Despite many studies investigating visfatin relation to periodontal diseases, there was a possibility to meta-analysis a few of them.

Various factors can cause heterogeneity in study data. For instance, obesity and the fat profile of individuals can be associated with periodontal disease and visfatin level. Additionally, other factors such as age, gender, inflammatory conditions of the body, varying periodontal microbiomes in individuals, the sampling time concerning the stage of disease activity and sampling method can also affect this heterogeneity. However, in each study, the age range, gender, BMI, history of systemic disease, and clinical parameters of periodontal diseases were monitored. In addition, in the analyzes conducted, there was evidence of the presence of publication bias, which can be effective in the final conclusion. Therefore, it is recommended to use the results cautiously.

We suggest designing a study with a full classification of disease severity based on accurate clinical parameters and degree of inflammation and full consideration of influencing variables for statistical analysis of all these items. Also, various forms of periodontal disease with a higher number of individuals be considered. Studying visfatin by PCR in biopsy of diseased periodontal tissue can give a clearer insight into the role of visfatin in pathogenesis of periodontal disease.

## Conclusion

Adipocytokine visfatin has an increased level in periodontal diseases, which plays an influential role in the inflammatory process of these diseases. Visfatin levels of GCF, serum, and saliva can have the potential to be used as a diagnostic biomarker of periodontitis as they are correlated with periodontal clinical parameters. Further study is recommended in this regard to ensure its specificity and sensitivity.

## Supporting information

S1 Checklist PRISMA 2009 checklist. (DOC)

S1 Table PubMed, Web of Science (ISI), and Scopus search strategy for systematic review and meta-analysis of visfatin level in periodontal disease. (DOCX)

S2 Table A summary of characteristics of the studies that were excluded from the meta-analysis. (DOCX)

S3 Table Risk of bias within studies. Newcastle-Ottawa assessment scale for case-control studies.
(DOCX)
