# Supplementary material for: Periodontal disease and visfatin level: A systematic review and meta-analysis
Source: PLoS One. 2023 Nov 7;18(11):e0293368. doi: 10.1371/journal.pone.0293368 (PMC10629655; doi:10.1371/journal.pone.0293368)
Supplement: S1 Table — (DOCX) [file pone.0293368.s002.docx]

Study of Visfatin level in periodontal disease: A systematic review and meta-analysis

S1A: History and Search Details (PubMed) date: March 12, 2023

| Search | Query |
| --- | --- |
| #3 | Search: ("nicotinamide phosphoribosyltransferase"[Mesh] OR "Nicotinamide Phosphoribosyltransferase" [tw] OR Visfatin[tw] OR "NAMPT Protein"[tw] OR "Pre-B-Cell Colony-Enhancing Factor"[tw] OR "Colony-Enhancing Factor, Pre-B-Cell"[tw] OR "Pre B Cell Colony Enhancing Factor"[tw] OR "NAmPRTase"[tw] OR "NMN Pyrophosphorylase"[tw] OR "nicotinamide phosphoribosyltransferase, human" [Supplementary Concept]) AND ("Periodontal Diseases"[Mesh] OR "Periodontal Diseas*"[tw] OR "Periodontitis"[Mesh] OR "Periodontitis"[tw] OR "Chronic Periodontitis"[Mesh] OR "Chronic Periodontitis"[tw] OR "gingivitis"[Mesh] OR Gingivitis[tw] OR "periodontal index"[Mesh] OR Periodontal Index[tw] OR "aggressive periodontitis"[Mesh] OR "Aggressive Periodontitis"[tw] OR "periodontal inflammation"[tw]) Sort by: Most Recent |
| #2 | Search: "nicotinamide phosphoribosyltransferase"[Mesh] OR "Nicotinamide Phosphoribosyltransferase" [tw] OR Visfatin[tw] OR "NAMPT Protein"[tw] OR "Pre-B-Cell Colony-Enhancing Factor"[tw] OR "Colony-Enhancing Factor, Pre-B-Cell"[tw] OR "Pre B Cell Colony Enhancing Factor"[tw] OR "NAmPRTase"[tw] OR "NMN Pyrophosphorylase"[tw] OR "nicotinamide phosphoribosyltransferase, human" [Supplementary Concept] Sort by: Most Recent |
| #1 | Search: "Periodontal Diseases"[Mesh] OR "Periodontal Diseas*"[tw] OR "Periodontitis"[Mesh] OR "Periodontitis"[tw] OR "Chronic Periodontitis"[Mesh] OR "Chronic Periodontitis"[tw] OR "gingivitis"[Mesh] OR Gingivitis[tw] OR "periodontal index"[Mesh] OR Periodontal Index[tw] OR "aggressive periodontitis"[Mesh] OR "Aggressive Periodontitis"[tw] OR "periodontal inflammation"[tw] Sort by: Most Recent |

S1B: Search History in ISI: date: March 12, 2023

| **Set** | **Save History / Create AlertOpen Saved History** |
| --- | --- |
| # 3 | #2 AND #1  *Indexes=SCI-EXPANDED, SSCI, A&HCI, CPCI-S, CPCI-SSH, BKCI-S, BKCI-SSH, ESCI Timespan=All years* |
| # 2 | **TOPIC:** ("nicotinamide phosphoribosyltransferase") *OR* **TOPIC:** (Visfatin) *OR* **TOPIC:** ("NAMPT Protein") *OR* **TOPIC:** ("Pre-B-Cell Colony-Enhancing Factor") *OR* **TOPIC:** ("Colony-Enhancing Factor, Pre-B-Cell") *OR* **TOPIC:** ("Pre B Cell Colony Enhancing Factor") *OR* **TOPIC:** ("NAmPRTase") *OR* **TOPIC:** ("NMN Pyrophosphorylase")  *Indexes=SCI-EXPANDED, SSCI, A&HCI, CPCI-S, CPCI-SSH, BKCI-S, BKCI-SSH, ESCI Timespan=All years* |
| # 1 | **TOPIC:** ("Periodontal Diseas*") *OR* **TOPIC:** ("Periodontitis") *OR* **TOPIC:** ("Chronic Periodontitis") *OR* **TOPIC:** ("gingivitis") *OR* **TOPIC:** ("periodontal index") *OR* **TOPIC:** ("aggressive periodontitis") *OR* **TOPIC:** ("periodontal inflammation")  *Indexes=SCI-EXPANDED, SSCI, A&HCI, CPCI-S, CPCI-SSH, BKCI-S, BKCI-SSH, ESCI Timespan=All years* |
|  |  |

**S1C:** Scopus Search on March 12, 2023

| History Count | Search Terms |
| --- | --- |
| 3 | ( ( TITLE-ABS-KEY ( "nicotinamide phosphoribosyltransferase" )  OR  TITLE-ABS-KEY ( visfatin )  OR  TITLE-ABS-KEY ( "NAMPT Protein" )  OR  TITLE-ABS-KEY ( "Pre-B-Cell Colony-Enhancing Factor" )  OR  TITLE-ABS-KEY ( "Colony-Enhancing Factor, Pre-B-Cell" )  OR  TITLE-ABS-KEY ( "Pre B Cell Colony Enhancing Factor" )  OR  TITLE-ABS-KEY ( "NAmPRTase" )  OR  TITLE-ABS-KEY ( "NMN Pyrophosphorylase" ) ) )  AND  ( ( TITLE-ABS-KEY ( "Periodontal Diseas*" )  OR  TITLE-ABS-KEY ( periodontitis )  OR  TITLE-ABS-KEY ( "Chronic Periodontitis" )  OR  TITLE-ABS-KEY ( gingivitis )  OR  TITLE-ABS-KEY ( "periodontal index" )  OR  TITLE-ABS-KEY ( "aggressive periodontitis" )  OR  TITLE-ABS-KEY ( "periodontal inflammation" ) ) ) View Less |
| 2 | ( TITLE-ABS-KEY ( "Periodontal Diseas*" )  OR  TITLE-ABS-KEY ( periodontitis )  OR  TITLE-ABS-KEY ( "Chronic Periodontitis" )  OR  TITLE-ABS-KEY ( gingivitis )  OR  TITLE-ABS-KEY ( "periodontal index" )  OR  TITLE-ABS-KEY ( "aggressive periodontitis" )  OR  TITLE-ABS-KEY ( "periodontal inflammation" ) ) |
| 1 | ( TITLE-ABS-KEY ( "nicotinamide phosphoribosyltransferase" )  OR  TITLE-ABS-KEY ( visfatin )  OR  TITLE-ABS-KEY ( "NAMPT Protein" )  OR  TITLE-ABS-KEY ( "Pre-B-Cell Colony-Enhancing Factor" )  OR  TITLE-ABS-KEY ( "Colony-Enhancing Factor, Pre-B-Cell" )  OR  TITLE-ABS-KEY ( "Pre B Cell Colony Enhancing Factor" )  OR  TITLE-ABS-KEY ( "NAmPRTase" )  OR  TITLE-ABS-KEY ( "NMN Pyrophosphorylase" ) ) |
