# Supplementary material for: Periodontal disease and visfatin level: A systematic review and meta-analysis
Source: PLoS One. 2023 Nov 7;18(11):e0293368. doi: 10.1371/journal.pone.0293368 (PMC10629655; doi:10.1371/journal.pone.0293368)
Supplement: S2 Table — (DOCX) [file pone.0293368.s003.docx]

**S2 Table. A summary of characteristics of the studies that were excluded from the meta-analysis.**

| ID | Author | Year | Country | Study | Reason for exclusion from meta-analysis | Case group | Visfatin analysis method | Results |
| --- | --- | --- | --- | --- | --- | --- | --- | --- |
|  | Tabari Z.A | 2015 | Iran | RCT | Reiterative study with Tabari ZA (2014) |  |  |  |
|  | Moazen M | 2022 | Iran | cross-sectional study | No English full text | Chronic periodontitis | GCF | Positive association of GCF visfatin concentration with endometrial cancer and CP |
|  | Chen F | 2018 | China | cross-sectional study | No English full text | Chronic Periodontitis | serum | positive correlation between CP and visfatin level  visfatin may have a role to play in pathogenesis of periodontitis |
|  | Yu T.H | 2017 | Taiwan | cross-sectional study | Different periodontal evaluation | Periodontal disease | serum | elevated plasma levels of visfatin in PD patients with CAD, suggesting a close relationship between visfatin and chronic inflammation |
|  | MiShra V | 2016 | India | cross-sectional study | Clinical attachment loss not examined | Chronic Periodontitis | GCF | Higher visfatin level in CP and CP-T2DM subjects. visfatin may have a role to play in pathogenesis of periodontitis |
|  | Mohamed H. G | 2015 | Sudan | cross-sectional study | Clinical attachment loss not examined | Chronic Periodontitis | GCF | significant positive correlation between CP and visfatin |
|  | Ghallab NA | 2015 | Egypt | cross-sectional study | Different visfatin sample | Choronic periodontitis | Biopsy  (ELISA visfatin)  (mRNA PCR) | Higher visfatin mRNA and its expression in T2DM+CP group and CP group  visfatin may have role in the pathogenesis of CP and T2DM |
|  | Ozcan E | 2017 | Turkey | cross-sectional study | Different visfatin sample | Choronic periodontitis | Biopsy  (Immunohistochemical detection of visfatin & mRNA PCR) | Higher visfatin expression in CP tissues  Increased visfatin was associated with the expression of NF-κB and PI3k which may play a role in the pathogenesis of periodontitis |
|  | Tabari ZA | 2018 | Iran | cross-sectional study | Different visfatin sample | Choronic periodontitis  Aggressive periodontitis | Biopsy  (Immunohistochemical detection of visfatin) | Higher visfatin expression in both CP and AP group with no difference between them  Positive relationship between inflammation grading and visfatin expression in AP |
|  | Isler SC* | 2021 | Turkey | cross-sectional study | Different visfatin sample | Periodontitis  Peri-implantitis  Periodontitis+Peri-implantitis | Biopsy  (Immunohistochemical detection of visfatin)  (mRNA PCR) | Higher visfatin mRNA expression levels in Periodontitis  Higher visfatin expression in Periodontitis and Peri-implantitis |
|  | Ozcan E | 2016 | Turkey | cross-sectional study | No quantitative data  (Median reported) | Periodontitis | GCF | Higher GCF visfatin levels in patients with periodontitis  The GCF visfatin levels had a positive correlation with clinical periodontal parameters |
|  | Ozcan E | 2015 | Turkey | cross-sectional study | No quantitative data  (Median reported) | Gingivitis  Periodontitis | saliva | Higher salivary visfatin levels in gingivitis and periodontitis  No significant difference was found between the gingivitis and the periodontitis groups |
|  | Türer C | 2016 | Turkey | RCT | No quantitative data | Gingivitis  Periodontitis | GCF  serum | Higher GCF and serum visfatin levels in gingivitis and periodontitis  Significant difference between the gingivitis and the periodontitis groups  Serum levels are consistent with visfatin levels detected in GCF |
|  | Shalaby HK* | 2020 | Egypt | RCT | - | Periodontitis | GCF | Higher visfatin level in periodontitis with T2DM and those with periodontitis only |
|  | Kemer Doğan E. S* | 2022 | Turkey | cross-sectional study | No quantitative data | Periodontitis | Saliva  serum | Higher visfatin levels in periodontitis groups  Positive relationships between salivary/serum visfatin levels and periodontal parameters and periodontal inflamed surface area (PISA) and obesity |
|  | Omer B | 2016 | Iraq | cross-sectional study | Unclear measurement unit of visfatin | Chronic periodontitis | Saliva (ELISA) | positive correlation between visfatin levels and PPD and CAL scores  Creatine Kinase and visfatin can be used as a marker of periodontitis and coronary atherosclerosis. |
|  | Bahammam MA | 2018 | Saudi Arabia | cross-sectional study | Unclear measurement unit of visfatin | Chronic periodontitis | GCF(ELISA) | Higher visfatin level in CP and CP-T2DM subjects  Periodontal destruction and diabetes have a synergistic effect on the elevation of inflammatory cytokine levels |

*Studies classified periodontal disease based on the 2018 classification and others are based on the 1999 classification. CAD= Coronary artery disease; PD= Periodontal disease: T2DM= Type 2 Diabetes Mellitus; RCT= Randomized controlled clinical trial; GCF= Gingival Crevicular Fluid; CP= Chronic periodontitis; AP= Aggressive periodontitis; PPD= probing pocket depth; ELISA= Enzyme-linked immunosorbent assay; mRNA= messenger ribonucleic acid; PCR= Polymerase Chain Reaction.
