# Supplementary material for: Periodontal disease and visfatin level: A systematic review and meta-analysis
Source: PLoS One. 2023 Nov 7;18(11):e0293368. doi: 10.1371/journal.pone.0293368 (PMC10629655; doi:10.1371/journal.pone.0293368)
Supplement: S3 Table — Newcastle-Ottawa assessment scale for case-control studies. (DOCX) [file pone.0293368.s004.docx]

**S3 Table. Risk of bias within studies.**

| Study | **Selection** | | | | **Comparability** | **Exposure** | | | **Total** |
| --- | --- | --- | --- | --- | --- | --- | --- | --- | --- |
|  | Definition of case | Represent-ativeness of cases | Selection of controls | Definition of controls |  | Assessment of exposure | Same methods of ascertainment for cases and controls | Non response rate |  |
| Banna SE [25] | + | + | - | - | ++ | + | + | - | 6 |
| Cetiner D [28] | + | + | - | + | ++ | + | - | - | 6 |
| Coutinho A [34] | + | + | - | + | ++ | + | + | + | 8 |
| Kadkhodazadeh M [26] | + | - | - | + | ++ | + | + | - | 6 |
| Mopidevi A [29] | + | + | - | + | ++ | + | + | + | 8 |
| Ozcan E [27] | + | + | - | + | ++ | + | + | - | 7 |
| Paul R [32] | + | + | - | + | ++ | + | + | + | 8 |
| Pradeep AR [16] | + | + | - | + | ++ | + | + | - | 7 |
| Pradeep AR [17] | + | + | - | + | ++ | + | + | + | 8 |
| Raghavendra NM [24] | + | + | - | + | ++ | + | + | + | 8 |
| Rezaei M [30] | + | + | - | + | ++ | + | + | + | 8 |
| Saljoughi F [33] | + | + | - | + | ++ | + | + | - | 7 |
| Saseendran G [35] | + | + | - | + | ++ | + | + | + | 8 |
| Surya D [31] | + | + | - | + | ++ | + | + | + | 8 |
| Tabari ZA [19] | + | + | - | + | ++ | + | + | + | 8 |
| Xu T.-H [36] | + | + | - | + | ++ | + | + | - | 7 |

Newcastle-Ottawa assessment scale for case-control studies.
